# Devastating delayed cerebral ischemia after aneurysmal subarachnoid hemorrhage

**DOI:** 10.3389/fneur.2022.1016111

**Published:** 2022-10-13

**Authors:** Inez Koopman, Philippine B. van Wijngaarden, Gabriel J. E. Rinkel, Mervyn D. I. Vergouwen

**Affiliations:** Department of Neurology and Neurosurgery, UMC Utrecht Brain Center, University Medical Center Utrecht, Utrecht University, Utrecht, Netherlands

**Keywords:** subarachnoid hemorrhage, delayed cerebral ischemia, blood pressure instability, coma, nimodipine

## Abstract

**Background:**

We investigated the proportion of patients in an initial good clinical condition who developed devastating DCI, and aimed to characterize these patients by aneurysm location, blood pressure instability prior to DCI, and the extent of cerebral ischemia.

**Methods:**

We included aSAH patients admitted between 2010 and 2021 with a Glasgow Coma Scale of 11 or higher 24 h after aneurysm treatment, who developed devastating DCI, defined as DCI leading to coma for at least 48 h with cerebral infarction on the subsequent scan. Blood pressure instability was defined as nimodipine-induced blood pressure drops, dosage adjustments, or the use of blood pressure drugs before onset of DCI. Descriptive statistics were used to summarize the data.

**Results:**

Out of 1,211 consecutive aSAH patients, 617 patients had a good clinical condition after aneurysm treatment of whom 16 (3%) patients [14 (88%) women] were included in this study. Thirteen (81%) patients had an aneurysm in the anterior circulation. Thirteen patients (81%) had blood pressure instability: twelve (75%) had nimodipine-induced blood pressure drops, eleven (69%) received antihypertensive drugs, and 7 (44%) received hypertension induction before onset of DCI. Thirteen (81%) patients had bilateral ischemia, mainly in the anterior circulation (56%).

**Conclusions:**

The proportion of aSAH patients with a good clinical condition after aneurysm treatment who develop devastating DCI is small. The vast majority of these patients had blood pressure instability. Future studies are needed to investigate if a reduction in the number and extent of blood pressure fluctuations decreases the incidence of devastating DCI.

## Introduction

Delayed cerebral ischemia (DCI) is an important determinant of poor functional outcome after aneurysmal subarachnoid hemorrhage (aSAH). Approximately 30% of aSAH patients develops DCI (1). The clinical course of DCI is highly variable. Symptoms may spontaneously improve or can be devastating with progression to coma and finally death. In aSAH patients in an initial good clinical condition there may be a window of opportunity to avert devastating DCI. We aimed to characterize devastating DCI in aSAH patients who were in good clinical condition after aneurysm treatment by aneurysm location, blood pressure instability prior to DCI, and extent of cerebral ischemia.

## Methods

### Study population

The Institutional Review Board of the University Medical Center Utrecht (UMC Utrecht) waived individual patient consent and formal ethics approval for this study, since we used data available from routine patient care. We performed a retrospective analysis of consecutive aSAH patients admitted to the UMC Utrecht between January 1, 2010 and April 5, 2021. Inclusion criteria were: (1) aneurysm treatment ≤72 h after ictus; (2) a Glasgow Coma Scale (GCS) score of 11 or higher 24 h after aneurysm treatment; (3) development of devastating DCI, which was defined as DCI leading to coma (GCS ≤8) lasting for at least 48 h with confirmation of cerebral infarction on subsequent head computed tomography (CT) or magnetic resonance imaging (MRI) (2); and (4) onset of devastating DCI within 14 days after ictus. Intubated patients were included if 24 h after aneurysm treatment eye opening was spontaneous or in response to verbal command, in combination with obeying commands. Patients in the intervention arm of the HIMALAIA trial (induced hypertension for DCI) were excluded (3). Our local treatment protocol for prevention and treatment of DCI is described in the Supplementary material.

### Data collection

The following characteristics were retrieved: age, sex, loss of consciousness at onset, GCS score on admission and 24 h after aneurysm treatment, Prognosis on Admission of Aneurysmal Subarachnoid Hemorrhage (PAASH) score (4), Hijdra et al. (5) sum score for the amount of extravasated blood on the initial head CT, history of hypertension, smoking status, location of the ruptured aneurysm, aneurysm treatment modality, complications ≤14 days after ictus, nimodipine-induced blood pressure drops and dosage adjustments, prescription of other blood pressure drugs (antihypertensive drugs and hypertension induction), and modified Rankin Scale (mRS) score 3 months after ictus (definitions are described in the Supplementary material). Blood pressure instability was defined as nimodipine-induced blood pressure drops, nimodipine dosage adjustments, and the use of blood pressure drugs and evaluated until the onset of devastating DCI.

### Statistical analysis

Data were summarized using descriptive statistics.

## Results

**Figure 1:**
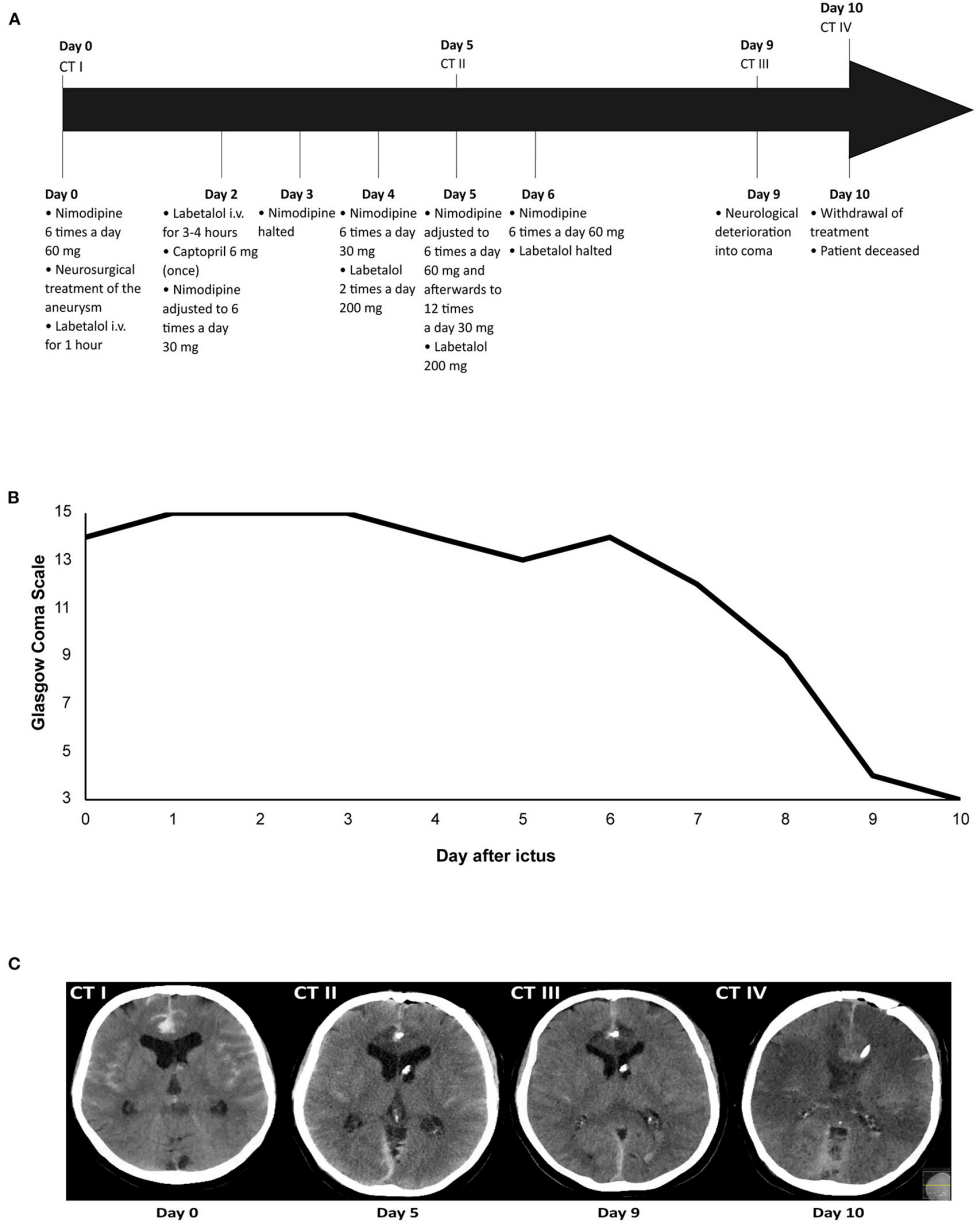
Illustrative case of a patient with devastating DCI. Time overview of: **(A)** Blood pressure medication adjustments and computed tomography (CT) scans; **(B)** Glasgow Coma Scale score; and **(C)** Ischemic lesions due to delayed cerebral ischemia on CT. The patient was admitted on day 0 and received nimodipine 6 times a day 60 mg. After neurosurgical treatment of the aneurysm on day 0, labetalol i.v. was administered for 1 h due to a high blood pressure, after which it was halted. Administration of nimodipine 6 times a day 60 mg continued. On day 2, labetalol i.v. was restarted because of a high blood pressure, but after 3–4 h labetalol was tapered and halted. Captopril 6 mg was administered once to avert a rise in blood pressure after labetalol was halted. Administration of nimodipine 60 mg resulted in a diastolic blood pressure drop of ≥10 mmHg, after which nimodipine dosing was lowered to 6 times a day 30 mg. On day 3, nimodipine administration was halted because of a drop in diastolic blood pressure ≥10 mmHg. After nimodipine administration was halted for 12 h, it was restarted again on day 4 at a dosage of 6 times a day 30 mg. Because of persistent systolic blood pressures >200 mmHg on day 4, the patient received labetalol 2 times a day 200 mg. On day 5, nimodipine dosing was increased to 6 times a day 60 mg, after which it was adjusted to 12 times a day 30 mg due to a drop in diastolic blood pressure ≥10 mmHg. The patient also received labetalol 200 mg. On day 6, nimodipine dosing was increased again to 6 times a day 60 mg and labetalol was halted as it was decided to accept systolic blood pressures >200 mm Hg. On day 9, the patient deteriorated into coma due to delayed cerebral ischemia. The patient was intubated and transferred to the ICU. On day 10, the decision was made to withdraw treatment, after which the patient was extubated. The patient died on day 10 after ictus.

Of the 1,211 aSAH patients, 617 patients had a good clinical condition after aneurysm treatment of whom 17 (3%) developed devastating DCI (illustrative case Figure 1; Supplementary Figure 1). One patient was excluded due to participation in another study (hypertension induction for DCI) (3). Patient characteristics are shown in Tables 1, 2. Thirteen (81%) patients had an aneurysm in the anterior circulation. Thirteen patients (81%) had blood pressure instability. Twelve (75%) patients had nimodipine-induced blood pressure drops and in 9 (56%) patients nimodipine dosing was adjusted, reduced or temporarily halted. Eleven (69%) patients received antihypertensive drugs, while 7 (44%) received hypertension induction at any moment before neurological deterioration. Five (31%) patients received both antihypertensive drugs and hypertension induction (not related to DCI) during the clinical course, but not simultaneously. Thirteen (81%) patients developed bilateral cerebral infarction and three (19%) patients unilateral cerebral infarction, mainly in the anterior circulation (69 and 67%). Three months after ictus, 13 (81%) patients had died, 2 (13%) patients were severely disabled, and 1 (6%) patient was moderately severe disabled.

**Table 1:** Patient characteristics.

	**All patients *N* = 16 (%)**
Female sex	14 (88)
Mean age, years [SD]	55 [13]
PAASH score on admission*	
1 (GCS 15)	4 (25)
2 (GCS 11–14)	6 (38)
3 (GCS 8–10)	2 (13)
4 (GCS 4–7)	2 (13)
5 (GCS 3)	0 (0)
Loss of consciousness at onset	10 (62)
Median Hijdra sum score [IQR]	29 [24–31]
History of hypertension	5 (31)
**Smoking status**	
Current smoker	8 (50)
Past smoker	3 (19)
Never smoker	3 (19)
Unknown	2 (13)
Anterior circulation aneurysm	13 (81)
**Aneurysm treatment modality**	
Endovascular coiling	5 (31)
Neurosurgical clipping	11 (69)
Occurrence of complications ≤ 14 days after ictus	14 (88)
Rebleeding	4 (25)
Hydrocephalus	9 (56)
Infection	7 (44)
Nimodipine-induced blood pressure drops^†^	12 (75)
Nimodipine dosing adjusted, reduced or temporarily discontinued	9 (56)
Use of antihypertensive drugs other than nimodipine	11 (69)
IV antihypertensive drugs	11 (69)
Oral antihypertensive drugs	8 (50)
Use of hypertension induction not related to DCI	7 (44)
Noradrenaline	6 (38)
Phenylephrine	3 (19)
Subsequent use of antihypertensive drugs (other than nimodipine) and hypertension induction	5 (31)
**Extent of cerebral infarction**	
Unilateral	3 (19)
Anterior circulation	2 (67)
Anterior and posterior circulation	1 (33)
Bilateral	13 (81)
Anterior circulation	9 (69)
Anterior and posterior circulation	4 (31)
**mRS score 3 months after ictus**	
Moderately severe disability	1 (6)
Severe disability	2 (13)
Death	13 (81)

*PAASH score could not be determined in two intubated patients;^†^Defined as: (1) a drop ≥20% in the MAP after nimodipine administration; or (2) a diastolic blood pressure drop ≥10 mmHg within 2 h of nimodipine administration. SD, standard deviation; GCS, glasgow coma scale; IQR, interquartile range; IV, intravenous; and Mrs, modified rankin scale.

**Table 2:** Individual patient characteristics.

**No**.	**Sex**	**Age**	**Aneurysm location**	**Type of aneurysm treatment**	**Blood pressure instability**						Day of DCI onset*	Day of death**	**WOC**	**Cause of death**	**Infections**
					**Nimodipine-induced blood pressure drops**	**Nimodipine dosage adjustments**	**Anti-hypertensive drugs**	**Specification**	**Hypertension induction not related to DCI**	**Specification**					
1	F	46	Right MCA	Neurosurgical clipping	+	+	-		+	4 days noradrenaline	11	79	No	Unknown (transfer to other hospital)	
2	F	73	ACOM	Endovascular coiling	+	+	+	1 day furosemide	+	6 days noradrenaline	9	n/a	Yes	n/a	Pneumonia
3	F	41	ACOM	Neurosurgical clipping	-	-	-		-		8	14	Yes	DCI	
4	F	47	ACOM	Neurosurgical clipping	+	-	+	2 days labetalol IV 1 day clonidine IV	-		9	10	Yes	DCI	Pneumonia
5	F	66	Left pericallosal artery	Neurosurgical clipping	-	-	-		-		11	16	Yes	DCI	
6	F	57	Basilar artery	Endovascular coiling	+	-	+	1 day labetalol IV 6 days perindopril	-		7	n/a	No	n/a	
7	M	47	ACOM	Neurosurgical clipping	+	+	+	1 day labetalol IV 3 days a combination of perindopril, amlodipine, metoprolol and clonidine	+	2 days noradrenaline	10	11	Yes	DCI	
8	F	53	Right vertebral artery	Neurosurgical clipping	+	+	+	1 day labetalol IV 1 day clonidine	+	1 day phenylephrine	5	12	Yes	DCI	
9	F	31	Basilar artery	Endovascular coiling	-	-	+	1 day labetalol IV	-		7	9	Yes	DCI	Meningitis (not culture-proven)
10	F	61	Left MCA	Neurosurgical clipping	+	+	+	3 days labetalol IV	+	1 day noradrenaline 1 day phenylephrine	10	39	Yes	DCI	Urinary tract infection Meningitis (not culture-proven)
11	M	81	ACOM	Neurosurgical clipping	+	+	+	2 days metoprolol	+	2 days noradrenaline 2 days phenylephrine	8	10	Yes	DCI	
12	F	38	ACOM	Neurosurgical clipping	+	+	+	1 day labetalol IV	-		8	10	Yes	DCI	
13	F	52	ACOM	Endovascular coiling	-	-	-		-		10	n/a	No	n/a	S. aureus bacteremia and endocarditis
14	F	62	ACOM	Neurosurgical clipping	+	+	-		+	1 day noradrenaline	9	25	Yes	DCI	Pneumonia
15	F	55	ACOM	Endovascular coiling	+	-	+	4 days labetalol IV	-		3	5	Yes	DCI	Pneumonia
16	F	65	Left pericallosal artery	Neurosurgical clipping	+	+	+	4 days labetalol IV	-		9	10	Yes	DCI	

*In days (ictus = day 0). **In days (ictus = day 0) within 3 months after ictus. F, female; M, male; MCA, middle cerebral artery; ACOM, anterior communicating artery; DCI, delayed cerebral ischemia; WOC, withdrawal of care.

## Discussion

The proportion of aSAH patients with a good clinical condition after aneurysm treatment who develop devastating DCI is small. Most of these patients had blood pressure instability during hospital stay before onset of devastating DCI.

Several previous studies investigated the relationship between nimodipine, blood pressure, and DCI (6–9). In one study, all patients who developed DCI had an impaired cerebral autoregulation (6). In other studies, DCI was associated with increased systolic blood pressures (SBP) during the first days, and a larger fall in SBP after nimodipine administration compared to patients without DCI (7, 8). Nimodipine dosage reductions or discontinuations occurred in half of the patients with cerebral infarction, which is in line with the observations in our study (9).

Several hypotheses have been postulated to explain the pathogenesis of DCI, including macrovascular spasm, microvascular spasm, microthrombosis, impaired cerebral autoregulation, inflammation, and cortical spreading ischemia. The blood pressure instability we observed in our patients prior to the onset of DCI support the hypothesis of an impaired cerebral autoregulation. However, whether such an impaired cerebral autoregulation may lead to repetitive ischemic insults to the brain or whether it is a manifestation of DCI remains to be investigated.

Some limitations need to be addressed. First, this was a retrospective analysis of clinical data from prospectively identified patients. Data were therefore not documented in a standardized way. As a result, there were missing data on the extent of blood pressure drops after the administration of nimodipine or antihypertensive drugs. Second, administration of analgesics may have occurred simultaneously with nimodipine administration and therefore influenced blood pressure values. However, most nimodipine-induced blood pressure drops occurred multiple times during admission, making it less likely that these drops were caused by analgesics. Third, this study is an exploratory study without a comparator group. Results should therefore be validated in a case-control study.

In conclusion, most patients with devastating DCI had blood pressure instability. Future studies are needed to investigate if a reduction in the number and extent of blood pressure fluctuations decreases the incidence of devastating DCI.

## Data availability statement

Data will be shared upon reasonable request to the corresponding author after signing a Data Transfer Agreement.

## Ethics statement

The Institutional Review Board of the University Medical Center Utrecht (UMC Utrecht) waived individual patient consent and formal ethics approval for this study, since we used data available from routine patient care.

## Author contributions

IK and PW contributed to the study concept/design, data collection, data- and statistical analysis, interpretation of data, and drafting of the manuscript. MV and GR contributed to the study concept and design, data collection, interpretation of data, manuscript revision, and provided study supervision. All authors contributed to the article and approved the submitted version.

## Funding

This study was funded by a combined grant from the Netherlands Organization for Health Research and Development and the Dutch Brain Foundation (Grant Number: 95105015). MV was financially supported by a personal grant from the Dutch Heart Foundation (Clinical Established Investigator grant 2018T076).

## Conflict of interest

The authors declare that the research was conducted in the absence of any commercial or financial relationships that could be construed as a potential conflict of interest.

## Publisher's note

All claims expressed in this article are solely those of the authors and do not necessarily represent those of their affiliated organizations, or those of the publisher, the editors and the reviewers. Any product that may be evaluated in this article, or claim that may be made by its manufacturer, is not guaranteed or endorsed by the publisher.
