# Supplementary material for: Devastating delayed cerebral ischemia after aneurysmal subarachnoid hemorrhage
Source: Front Neurol. 2022 Oct 13;13:1016111. doi: 10.3389/fneur.2022.1016111 (PMC9606612; doi:10.3389/fneur.2022.1016111)
Supplement: Supplementary file 1 [file Data_Sheet_1.docx]

**Supplemental material**

Local treatment protocol for prevention and treatment of delayed cerebral ischemia p. 2
after aneurysmal subarachnoid hemorrhage
Definition complications p. 4
Figure S1. Patient flowchart p. 5
References p. 6

**Local treatment protocol for prevention and treatment of delayed cerebral ischemia after aneurysmal subarachnoid hemorrhage**
*Admission of aneurysmal subarachnoid hemorrhage patients*
Patients with aneurysmal subarachnoid hemorrhage (aSAH) are admitted to an intensive care unit (ICU) if the patient has a Glasgow Coma Scale (GCS) score of 8 or lower on admission with a need of mechanical ventilation. All other aSAH patients are admitted to a medium care unit (MCU). During admission, patients are admitted to the ICU if indicated, for example because of respiratory failure. If the ruptured aneurysm is treated by neurosurgical clipping, the patient is observed afterwards at the ICU for at least one day.

*Prevention and treatment of delayed cerebral ischemia*

All patients receive nimodipine 60 mg every 4 hours orally or via nasogastric tube. Nimodipine-induced blood pressure drops are defined according to our local treatment protocol. At the ICU, nimodipine-induced blood pressure drops are defined as: a drop ≥ 20% in the mean arterial pressure (MAP) after nimodipine administration. At the MCU, drops are defined as: a diastolic blood pressure (DBP) drop ≥ 10 mmHg within 2 hours after nimodipine administration. If nimodipine-induced blood pressure drops occur at the ICU, nimodipine dosing is adjusted to 12 times 30 mg. In case blood pressure drops remain with the adjusted dosing, nimodipine is temporarily halted and if possible resumed in a later phase. If blood pressure drops occur at the MCU, the dose is adjusted from 6 times a day 60 mg to 6 times a day 30 mg. If during the next 24 hours no blood pressure drops occur, nimodipine dosage is increased again to 6 times a day 60 mg. If blood pressure drops occur with 30 mg, administration of nimodipine is halted. After a temporary stop of 12 hours, nimodipine administration is resumed 6 times a day 30 mg and increased if possible. This process is repeated until the standard dose of nimodipine can be administered without blood pressure drops ≥ 10 mmHg. In addition to treatment with nimodipine, we aim for normovolemia (fluid intake 2.5-3L) with saline fluids (sodium chloride 0.9%). If clinical symptoms suggestive of delayed cerebral ischemia (DCI) occur, 500cc additional saline fluid is administered as soon as possible and subsequently a computed tomography angiography (CTA) and CT perfusion of the head is performed.

*Blood pressure regulation*

At the ICU, the intensivist aims for a MAP of 80-120 mmHg in comatose or sedated patients. In patients who are awake, lower blood pressures are accepted as long as this does not lead to neurological deterioration. Hypertension (a systolic blood pressure >180 mmHg in patients with an unsecured aneurysm, or >220 mmHg or a MAP >130 in patients with a secured aneurysm) is treated with analgesics or other medication if the patient is in pain or agitated. If the patient is not in pain or agitated, hypertension is treated with antihypertensive drugs unless this will lead to organ dysfunction. If antihypertensive drugs are administered, the aim is to achieve a gradual decrease in blood pressure with a maximum reduction of 10% in 2 hours. If a patient has a MAP <80 mmHg, an optimal fluid balance is the first method of treatment. Second method of treatment is noradrenaline. Hypertension induction is not used as a treatment for DCI. At the MCU, the use of antihypertensive drugs is avoided as much as possible and is only administered according to the treating physician’s opinion. Rescue therapy (induction of hypertension with vasopressor drugs, intra-arterial balloon angioplasty, intra-arterial treatment with vasodilator drugs) is not routinely used for prevention or treatment for DCI in our hospital, because there is no or low to moderate quality evidence for improved long-term outcome with these treatments.[1–5]

**Definition complications**
Rebleeding: A sudden clinical deterioration of the patient with signs of increased hemorrhage on the subsequent head-CT.
Devastating delayed cerebral ischemia: DCI leading to coma (Glasgow Coma Scale score ≤8) lasting for at least 48 hours with confirmation of cerebral infarction on subsequent head-CT or magnetic resonance imaging (MRI).
Hydrocephalus: A decrease in consciousness of the patient with enlargement of the ventricular system on head-CT.
Infection: Defined according to the criteria from the Center for Disease Control.[6]

**Figure S1.** Patient flowchart

Devastating DCI (n=16)

- Other causes (e.g. AVM, dissection) (n = 208)
- No cause identified (n = 173)

Devastating DCI (n=17)

- Aneurysm not treated (n = 184)
- Aneurysm not treated ≤72 hours after ictus (n = 199)
- GCS score <11 after aneurysm treatment (n=126)
- No follow-up data available for the first 14 days after ictus (n=85)

SAH (n= 1592)

aSAH (n= 1211)

aSAH with a good clinical condition after aneurysm treatment (n = 617)

- No DCI or no devastating DCI (n=544)
- Deterioration into coma from a cause other than DCI (n= 56)

- Included in the intervention arm of the HIMALAIA trial (n=1)

(a)SAH= (aneurysmal) subarachnoid hemorrhage; AVM= arteriovenous malformation; GCS= Glasgow Coma Scale; DCI= delayed cerebral ischemia HIMALAIA = Induced hypertension for delayed cerebral ischemia after aneurysmal subarachnoid hemorrhage.[1]

**References**

1. Gathier CS, van den Bergh WM, van der Jagt M, Verweij BH, Dankbaar JW, Müller MC, et al. Induced hypertension for delayed cerebral ischemia after aneurysmal subarachnoid hemorrhage: a randomized clinical trial. Stroke. 2018;49:76–83.

2. Venkatraman A, Khawaja AM, Gupta S, Hardas S, Deveikis JP, Harrigan MR, et al. Intra-Arterial vasodilators for vasospasm following aneurysmal subarachnoid hemorrhage: A meta-Analysis. J Neurointerv Surg. 2018;10:380–6.

3. Jun P, Ko NU, English JD, Dowd CF, Halbach VV, Higashida RT, et al. Endovascular treatment of medically refractory cerebral vasospasm following aneurysmal subarachnoid hemorrhage. Am J Neuroradiol. 2010;31:1911–6.

4. Dankbaar JW, Slooter AJC, Rinkel GJE, van der Schaaf IC. Effect of different components of triple-H therapy on cerebral perfusion in patients with aneurysmal subarachnoid haemorrhage: A systematic review. Crit Care. 2010;14:1–10.

5. Jabbarli R, Pierscianek D, Rölz R, Oppong MD, Kaier K, Shah M, et al. Endovascular treatment of cerebral vasospasm after subarachnoid hemorrhage. Neurology. 2019;93:E458–66.

6. Horan TC, Andrus M, Dudeck MA. CDC/NHSN surveillance definition of health care-associated infection and criteria for specific types of infections in the acute care setting. Am J Infect Control. 2008;36:309–32.
